# Diphtheria

**Published:** 1862-12

**Authors:** Jas. M. Corse

**Affiliations:** Chairman Committee


### SELECTED.

### DIPHTHERIA.

EXTRACT PROCEEDINGS PHILADELPHIA COUNTY MEDICAL SOCIETY.

Jas. M. Corse, M. D., Chairman Com.

During the past year this disease has prevailed amongst us, and has, in quite a number of instances, proved fatal in spite of the medical means employed for its cure. In many of its features it closely resembles other diseases, while a few of its symptoms and its sometimes intractable character, have distinguished it from them and placed it in the light of a new disease. Its protean character has given rise to difference of opinion in medical circles, as to whether it is a variety or complication of known diseases, or an entirely new scourge. In the present state of learning, we are unable to determinately settle the question, and must prescribe for it according to the phenomena observed in each case.

History shows us that long since, even as far back as 1576, a disease of a fatal character was noticed, which was characterized by many of the symptoms of diphtheria, and later writers are willing to consider it the same as the one which now prevails so extensively.

Dr. Wittig writes he " has met with a number of cases of diphtheria, of which only two were fatal; a false membrane was formed like that in croup, which passed down into the larynx; the febrile symptoms were milder, but the suffering greater than in croup.

" The disease occurred sometimes in conjunction with scarlatina and sometimes independently. I did not employ blisters because I have known them in croup to produce a slough. Aq. chlorinata; tr. ferri chlor. and nit. arg. and sulph. cupri were applied to the fauces.

" In one of my fatal cases the urgent symptoms subsided a few hours before death, and the patient became conscious; notwithstanding I gave wine whey, beef tea, and quinine, the patient continued to sink, and at last expired."

Dr. Atkinson says diphtheria has in common with scarlatina every premonitory symptom; when fully developed, everything except the rash, and leaves precisely similar sequelæ, and finally, that "it would require stronger arguments and more observation of well-marked cases to convince him that these two names do not rightfully belong to one affection." Dr. Maris, Resident Physisian to the Philadelphia Dispensary, says of diphtheria, "I am disposed to believe it may be induced by the poison of scarlatina, but that the diseases are identical I am not convinced. My plan of treatment consisted in the use of chlorate of potash, tr. ferri chlor., wine whey, beef tea, &c.; locally, gargle of dilute chlorinated soda, cupri, alum, &c."

Dr. Hamilton writes of diphtheria that "the symptoms are in some cases so similar to what we see in some cases of scarlatina and in croup, as lead to confusion and difficulty in diagnosis. Other cases were so decided and peculiar in feature, as to admit little doubt of their non-identity with scarlatina or croup."

Dr. Henry Hartshorne says: "My conviction of the non-identity of diphtheria with scarlatina, and of the pathological importance of the diagnosis between it and croup, has been strengthened by every case I have seen.

"This disease is one of the greatest importance in all respects, but more particularly from its extensive prevalence and fatality."

In reply to our circular, Dr. Condie has given a concise summary, which contains most of what may be said on the subject, and coming from one of his ripe experience and happy power of description, we deem it best to give his remarks on the subject entire.

Dr. Condie reports that, "compared with 1859, the past year, 1860, may, with propriety, be pronounced as a decidedly unhealthy one, throughout nearly every portion of the United States. It has been marked, certainly by the prevalence of more and severer disease, and a far greater amount of mortality than the year preceding.

"Various forms of catarrhal, bronchial and pneumonic diseases, were constantly met with during the spring, as well as during the latter portion of the summer, and from then throughout the autumn and winter up to the present time.

"During a portion of the year measles, chicken-pox, and a very malignant form of scarlatina prevailed, in different sections of the city, as somewhat extended epidemics. In certain neighborhoods a large number of cases of erysipelas and small-pox, with some of hooping cough, and many of child-bed fever. To this list of prevailing diseases must be added pseudo-membraneous angina or diphtheria. All of these still continue to show themselves to a greater or less extent, in many portions of the city.

"From the very commencement of 1859, diseases of the fauces and of the respiratory organs, varying much in intensity, were observed to occur more frequently than during preceding years. More especially was this the case in respect to inflammatory affections of the throat in persons of ages and conditions in life. Most of the cases which first occurred, were of simple sore throat—slight redness (with very little or no tumefaction) of the tonsils, of the palate, uvula, and often of the whole posterior portion of the pharynx, without, however, any diphtheric deposition, or ulceration. There was usually a sense of heat and dryness of the throat, some increase of soreness in swallowing, and a short, hacking, but not very frequent cough. These cases were attended with more or less, but never very intense or long continued, constitutional disturbance. They readily yielded to a very simple treatment, and seldom lasted beyond two or three days.

"Towards the close of spring, and during the damp and changeable weather of the summer, many cases were seen in which the affection of tonsils, palate and pharynx; greater intumescence of the mucous membrane--some difficulty in swallowing, and with the appearance, for the most part, upon the tonsils, and posterior and upper portion of the pharynx of very small patches of a dirty white, yellowish, or brownish exudation, very nearly circular in form, and with tolerably sharp and well defined edges. Sometimes the congested mucous membrane rising up around these patches, would give them the appearance of being depressed, as though the deposit was the bottom of a small ulcer. These cases were, like the former, in general, readily controlled, and usually terminated favorably at the end of four or five days, or at the furthest a week.

"Cases of genuine well-warked diphtheria became now extremely common in some sections of the city. The disease, however, as it presented itself in different patients, varied much in the intensity of the symptoms. In the simplest and mildest form it commenced without any very marked symptoms. The patient would occasionally complain of a sense of soreness, or of prickling about the fauces, with a slight degree of pain, extending along the Eustachian tube into the ear—some stiffness about the neck—dryness of the throat, a slight, dry cough, and more or less thirst. The tonsils were generally swollen, while the entire mucous membrane of the fauces was reddened, exhibiting considerable injection of its superficial vessels; the surface, at the same time, being uniformly copered with a thin, transparent, shining pellicle. The latter occasionally increased soon in density, so as to become opaque and acquire a whitish or yellowish hue. When this pellicle was detached the mucous membrane beneath was found to be without ulceration or abrasion. The tongue, particularly from about its centre towards its base, was more or less thickly indued with a dirty-white granular looking fur. By the fauces there was secreted a thick, tenacious, ropy fluid, not readily ejected from the mouth.

"This form of the disease was attended with but little danger, and seldom lasted, if it were properly managed, beyond a few days. Three-fourths of the cases seen by me were entirely well at the end of six or eight days. Occasionally, however, the case would suddenly assume, usually in its early stage, a much more severe and dangerous aspect—even acquiring, within a very short period, the most malignant features of the disease.

"In other cases, again, the attack was preceded for several days by a sense of languor and lassitude. Chills and flushes of heat would rapidly succeed each other. There would soon set in pain of the head, redness and suffusion of the eyes, stiffness and soreness of the neck, with intumescence and tenderness of the cervical glands. Pains extending into one or other of both ears, or frequently down the back and along the lower extremities were of common occurrence. There was always some difficulty of deglutition, which in severe cases was often so great as to cause, when an attempt was made to swallow fluids, a portion of these to be returned through the nose. The surface was hot and dry, the urine scanty and high-colored; often turbid and ropy. The bowels were mostly regular—though occasionally constipated. The appetite in some cases was entirely absent, while in others it was variable, or but slightly affected. Occasionally there was pain of the epigastrium, with nausea and vomiting, at intervals, of a glairy fluid tinged with bile; now and then every minute albuminated flocculi were seen floating in it.

"In some cases the attack was ushered in with a decided chill, succeeded by severe fever, which lasted usually about forty-eight hours, but, in many instances, it continued throughout the disease. For the most part, however, the febrile action was but slight. It was always more or less decidedly remittent, and often assumed from the onset a stronly marked adynamic type. In this more evidently febrile form of diphtheria, the throat, very early in the attack, became hot and sore. Deglutition, and, indeed, every movement of the muscles of the throat was attended with pain. The tongue was narrow and pointed, and thickly coated with a dirty white or yellowish pasty matter, through which the enlarged papilla were seen to protrude, in the form of red, shining points. The deposition upon the faucial mucous membrane was from an early period of the attack abundant and of a membraneous appearance.

"Its color was dirty white, yellowish or grayish. It usually commenced in small, distinct patches, which rapidly extended in size, and finally coalesced, so that soon the throat presented scarcely a single portion which was not uniformly coated. Sometimes the deposition took place simultaneously over nearly the whole of the throat. When it was separated from the faucial mucous membrane, the latter was found intact—or slightly reddened: occasionally, there was seen to ooze from its surface a blood serum, or even a drop or two of pure blood.

"In the great majority, if not in all the cases observed by me, the deposit in the throat was repeatedly thrown off and again renewed. In favorable cases it became thinner upon each renewal, until it ceased entirely to be formed.

"Even when the membraniform deposit was confined strictly to the tonsils, the palate or the pharynx, there was often observed some change in the tone, volume, and clearness of the patient's voice. There was, however, very little, if any, disturbance of respiration, so long as the deposit did not extend into the larynx. When, however, such extension occurred, the breathing became invariably difficult and stridulous, and there set in a loud ringing cough, as in croup; while the voice became low, whispering, or extinct.

"In a few cases there took place, even before any symptoms of severe illness became manifest, a rapid and most extensive deposit throughout every portion of the fauces, pharynx, and larynx, followed promptly by death from suffocation, as in the more acute form of membranous croup.

"In its more severe forms, the disease was apt to linger for ten or fifteen days, or even longer, with occasional changes, apparently for the better, but of short duration, the patient gradually sinking into a state of hopeless exhaustion. An attack of bronchitis of the most unmanageable form would not unfrequently occur, sometimes early in the attack. Pneumonia has, also, been observed, running a rapidly fatal course. It has happened, now and then, that after apparently the most complete convalescence had set in, a relapse would most unexpectedly ensue, followed shortly by a fatal termination. Even in severe cases of diphtheria—under what seemed at first sight the most unpromising circumstances—the disease after running a more protracted course—fluctuating for many days between better and worse—has been known eventually, under judicious management, to terminate favorably.

"Diphtheria assumed occasionally a form of very decided malignancy. The attack commenced in one or other of the manners above recited. Very early, however, indications of adynamia, of a more or less decided character, made their appearance. The strength of the patient became greatly depressed—there was a livid, dusky appearance of the countenance, and an acrid heat with great dryness of the surface. The entire throat presented, from the first, a thick coating of pultaceous, ash colored matter, which soon became of a dark brownish, and, finally, decidedly black hue, and exhaled a gangrenous odor. A fetid sanious fluid, of more or less consistence, was discharged from the nostrils. The cervical glands became greatly swollen, and often the whole of the anterior portion of the throat also, the integuments of which became tense and shining, and of a dusky red or mahogany color. In such cases, I have known suppuration to ensue, the matter being infiltrated throughout the cellular tissue of the parts affected, causing extensive sloughing, laying bare in a few instances the parotid glands, the trachea, and the large bloodvessels of the neck. The disease has been seen to extend from the throat in the posterior nares, involving in destruction all the soft parts from the roof of the pharynx, including the soft palate, to the base of the brain. In this malignant form of diphtheria the prognosis is always unfavorable—recovery from it being rather the exception than the rule.

"In all the forms of the disease an eruption upon the skin is liable to occur. It has been supposed to be confined chiefly to the more intense grades of the disease; I have observed it, however, as often in the mildest cases as in the most malignant. The eruption is sometimes slight and evanescent, but occasionally it is more generally diffused—of a more decided appearance, and continuing for several days. In these cases the eruption bears a close resemblance to that of measles—

inclinig to a very deep raspberry hue. In some instances, it was, however, the appearance simply of a slight efflloresence or scarlet rash—whenever this is the case we have found it to be very evanescent—or to come and go at short intervals throughout the entire continuance of the disease. This eruption cannot be said to be a very common accompaniment of diphtheria. In some of the epidemics of which we have the history, no mention is made of any eruption upon the skin having been observed; while in others it is described as constituting one of the prominent features of the disease. It is probable, however, that in many of these cases there was a combination of diphtheria with scarlet fever—both of which diseases have very generally prevailed in the same localities at one and the same time. This fact has been noticed in this country as well as in Europe.

"As sequelæ of diphtheria, I have observed abscess of the tonsils, parotids, and other glands of the throat and neck. Inflammation of one or both ears, followed by suppuration and a long-continued fetid discharge; a similar discharge from the nostrils is also liable to follow attacks of diphtheria. The discharge from the nose as well as that from the ear very generally caused a severe irritation of the parts over which it was allowed to flow. In some instances the attack of diphtheria was followed by a short, dry, obstinate, hacking cough —hoarseness—with loss of the natural tone and compass of voice—in a few cases with a total extinction of the voice. In other cases the sequelæ were chronic diarrhœa, intumescence of the face and extremities, sometimes of the entire body. In nearly every severe case convalescence was long and tedious, and the patient was liable throughout it to the occurrence of severe attacks of bronchitis or pneumonia, or even to die suddenly, after any unusual exertion, apparently, from mere exhaustion.

"Albuminuria was by no means a common occurrence in the cases of diphtheria, which have fallen under our notice, certainly in not more than one fourth. When it did occur, it made its appearance early in the attack. Its presence or absence did not appear to bear any relation to the severity or mildness of the attack, or to render the prognosis more unfavorable. The albuminous urine, even when most marked and profuse, would occasionally be found entirely free from tubal casts. Unlike what occurs in scarlatina, albumen has very seldom been detected in the urine during convalescence, never, unless it had been previously present.

"In by far the larger number of instances, which have fallen under my observation, the persons attacked with diphtheria were infants, and children under fifteen years of age. Occasionally, however, the disease was encountered in older patients—chiefly, within the range of my observation, females. The youngest patient with diphtheria treated by me was turned of two years—the oldest, a female, forty years of age.

"Diphtheria is evidently the product of some peculiar morbific condition of atmosphere, either confined to certain limited districts or, as usually takes place, spreading successively from district to district. Notwithstanding its epidemic features—its general disregard of the topographical character and the sanitary condition of the places visited by it—of the character and amount of their populations, or the season of the year, diphtheria has nevertheless been found to prevail most extensively, and with the greatest severity in low, damp, confined, unventilated, and otherwise unhealthy situations; during seasons marked by great and sudden vicissitudes of temperature, in conjunction with excessive atmospheric moisture, and among a crowded, squalid, badly-fed population, the members of which are regardless of personal and domestic cleanliness. I have not met with a single fact that would lead me to suspect that diphtheria is susceptible of being propagated by contagion. Whenever I have been able to investigate properly the circumstances which have been adduced in evidence of the contagious character of the disease, I have invariably found them to be simply cases in which the diphtheria has successively made its appearance in individuals who had visited or who were residents of a location in which it was at the time prevailing, and who, in consequence, were equally exposed to the same malaria, the same epidemic cause by which the disease was generated in those previously attacked, and from whom it was supposed to be contracted by those who sickened subsequently. In all these instances it was most certain that in those who took the disease it was generated by a common atmospheric cause, which, we admit, was more active in certain localities than in others within the sphere of the epidemic influence, from the want of due ventilation, overcrowding and other unfavorable hygienic conditions.

"As to the true pathological character of diphtheria: the common opinion formerly entertained that it is a specific inflammation of the mucous membrane of the throat, giving rise to a peculiar pellicular exudation upon the surface of the affected membrane without destruction of its integrity, will scarcely find any supporters at the present day. The entire history of the disease, its symptoms, progress and results, would seem to indicate its evident dependence upon some morbid condition of the general system—most probably arising from some abnormal condition of the blood. This view of its pathology seems to me to constitute the only true and safe basis for a rational and successful therapeutics. Whoever shall attempt the treatment of diphtheria, in its more severe forms at least, by remedies directed solely to the throat affection, will meet certainly with disappointment—a judicious and well managed constitutional treatment will alone be crowned with success. The efforts of the physician must be directed mainly to the restoration of the healthy condition of the blood—not, however, overlooking entirely the diseased condition of the throat. The removal of the membraniform deposit from the fauces, or the prevention of its accumulation and extension is always to be attended to. It is a measure calculated to give great relief to the patient, and will concur with the general treatment in rendering his recovery more prompt and certain.

"By some few writers on the disease diphtheria is set down as a mere form of scarlatina, unattended, for the most part, by the eruption on the surface. It is very certain that there is a very close affinity existing between diphtheria and scarlet fever, and, also, we believe it will be found, between it and epidemic erysipelas. All three of these diseases are liable to prevail together epidemically, in the same localities. They are all zymoses—their local and more prominent symptoms being all subordinate and secondary to a general morbid condition of the system. It is well known that diphtheritic deposition often occur in the throats of patients affected with scarlet fever, and we have seen that an eruption upon the skin is at least an occasional occurrence in connection with diphtheria; it is this, perhaps, which has led to the opinion that there is a perfect identity between the two diseases. We are convinced, however, that their diagnosis can always be very readily made out.

"During the past year, up to the present period, during the epidemic prevalence of diphtheria, the same thing has been observed as was observed in most—nearly all—of the former epidemics of the disease, that, namely, in conjunction with it, both scarlatina and erysipelas prevailed in the same neighborhoods, often in the same house. Cases of erysipelas, especially, have certainly fallen under our notice during the last eighteen months, more frequently and of a more severe and unmanageable character, than during the same length of time at any former period of our practice.

"In the treatment of the disease, so far at least as it fell under my notice, in the cases met with by me, I have been governed solely by the indications present in each case. In a few instances, where the disease occurred in young, robust, and plethoric habits, and was attended by decided febrile reaction and a hard or resisting, quick pulse, I have directed at the very onset of the attack, a moderate abstraction of blood by leeches applied to the neck in the neighborhood of the tonsils, and I am persuaded, with the very best results. In the majority of cases, however, direct depletion was strongly contra-indicated, and a tonic, cordial, and stimulant treatment demanded. My usual remedies were the tinct. of the muriate of iron, quinine, wine whey, milk punch, or even pure wine, or brandy and water, in doses, the size and repetition of which being adapted to the age of the patient and the urgency of the symptoms of adynamia present in each case. I have employed, and I believe with much advantage, the chloride of soda, by the stomach, and, locally, as a wash to the throat. The chlorinated mixture recommended by Dr. Watson, in his Lectures, as a remedy in scarlatina, has in my hands, also, proved decidedly beneficial. In many instances when the patient is seen early in the attack, some advise an emetic of sulphate of zinc to be given; we have seen, we think, much good result from the practice. The remedy with the effects of which I have been the most pleased, has been a combination of calomel, muriate of ammonia, ipecac. and extract of hyoscyamus—from 1 to 3 grains of the first, 2 to 4 grains of the second, $\frac{1}{4}$ to $\frac{1}{2}$ a grain of the third, and from $\frac{1}{4}$ to $\frac{1}{2}$ or even one grain of the fourth article, for a dose. To be repeated every two, three, or four hours. The dose, as well as the intervals of its repetition, to be regulated according to the age of the patient, the stage of the disease, and the urgency of the symptoms present.

"At the very commencement of the attack, when the diphtheritic deposit is small in amount, and confined chiefly to the anterior portion of the fauces, it is probable that a judicious application to the throat of nitrate of silver may have the effect of so modifying the morbid condition of the part as to prevent the extension of the disease and any very severe affection of the pharynx or larynx. As a general thing, how ever, I have not found the free and repeated use of the nitrate of silver, nor, indeed, of any of the caustic and stimulating applications to the throat so commonly prescribed in diphtheria to be productive of any decided good. In mild cases these applications have appeared to me to do positive harm, while in the more violent they do good, to say the least of them. In some instances, the application to the throat of the tincture of the muriate of iron, largely diluted with water, two or three times a day, or oftener, by means of a swab or brush, has appeared to me to be attended with beneficial results. I may make a similar remark in reference to a wash for the throat of diluted pyroligneous acid, or diluted hydrochloric acid and honey, or a strong solution of alum and honey. Gargles of a strong infusion of sage, rendered rough with alum and sweetened with honey—a saturated solution of borax and honey, or a solution of chloride of soda, one pint to sixteen of water, will often be found decidedly beneficial.

"Care should always be taken to supply the patient, at short, stated intervals, with good beef tea, chicken broth, new milk, cream, plain egg custard, oyster liquor with crumbs of cracker broken in it. The patient will often refuse to take food from, apparently, an inability, or perhaps a fear to swallow; by perseverance, however, and proper management he may, in general, be induced to take properly prepared nutriment up to even an advanced stage of the disease. During convalescence the diet should be improved as rapidly as possible, by the addition of the more easily digested meats, plainly cooked; with such vegetables as will be least liable to disturb the stomach or bowels.

"Cold water, as a means of quieting thirst, should be allowed, as well during the disease as during convalescence. It is peculiarly grateful to the patient, and a soothing application to the throat. Ice cream will often be taken, and has always appeared to me to do good by its coldness, and its lubricating properties, independently of the nutriment it furnishes.

"For a long time after convalescence has set in, it is all important to guard against cold and damp, and to protect him from any undue amount of exertion. His life even may be jeoparded by a neglect of this precaution."

